# The complete mitochondrial genome of the hybrid *Cyprinus capio Furong. [Cyprinus carpio L. mirror* (♀)×*Cyprinus Carpio var. singuonensis* (♂)]

**DOI:** 10.1080/23802359.2016.1180552

**Published:** 2016-06-22

**Authors:** Jinlong Wang, Li Zou, Guomin Jiang, Ming Zeng, Chuanwu Li, Yuanan Wu, Dongwu Wang, Mingqiu Liu, Li Liu

**Affiliations:** a Fisheries Research Institute of Hunan Province, Changsha, Hunan, P.R. China;; b Collaborative Innovation Center for Efficient and Health Production of Fisheries in Hunan Province, Changde, Hunan, P.R. China

**Keywords:** *Cyprinus capio Furong*, genome composition, mitogenome

## Abstract

The complete mitochondrial genome of the hybrid of *Cyprinus carpio L. mirror* (♀)× *Cyprinus Carpio var. singuonensis* (♂) was characterized first in this study. The total length of the genome was identical to the female parent as 16,581 bp, and the overall base composition was 31.80% A, 24.85% T, 27.55% C and 15.80% G, with a slight A + T bias. It contained 13 protein-coding genes (PCGs), 22 transfer RNA genes, 2 ribosomal RNA genes and 2 main non-coding regions (the control region and the origin of the light-strand replication). This study discovered the 99.70% sequence identity between the hybrid and its female parent, which confirmed the maternal inheritance pattern followed by the mitochondrial genome of the hybrid. However, the sequence alignment of mitochondrial genomes between the hybrid and its female parent revealed a total of 47 variable sites in 15 genes or regions, especially 25 sense mutations in 6 PCGs. The complete mitochondrial genome sequence of the hybrid *Cyprinus capio Furong* may provide an important dataset for further study in mitochondrial inheritance mechanism.

In animal evolution, hybridization is a powerful approach of inducing genetic and epigenetic alterations, leading to increased diversity and speciation (Barton 2008; Guan et al. 2015). A hybrid population of *Cyprinus carpio L. mirror* (♀) × *Cyprinus Carpio var. singuonensis* (♂) was obtained by artificial hybridization experiment for 40 years. The growth rate of the hybrid is 1.4 times of that of *Cyprinus carpio L. mirror* and 1.5 times of *Cyprinus Carpio var. singuonensis*. In this study, we obtained the hybrid population of *Cyprinus carpio L. mirror* (♀) × *Cyprinus Carpio var. singuonensis* (♂) complete mitochondrial genome. For a better understanding of the genetic status and the evolutionary study, we focused on the genetic information contained in the complete mitochondrial genomes of the fish.

The hybrid *Cyprinus capio Furong* was sampled randomly from Fisheries Research Institute of Hunan Province, and was deposited in the Museum of Fisheries Research Institute of Hunan Province (No. 218). We presented the complete mitochondrial DNA sequence of the hybrid *Cyprinus capio Furong* by the PCR-based method for the first time. The experimental and data analysis methods followed the previous study (Chu et al. 2013; Liu et al. 2015). The mitochondrial genome has been deposited in the GenBank with accession number KU146532. The mitochondrial genome which was 16,581 bp in length included 13 protein-coding genes (PCGs), 2 ribosomal RNA (rRNA) genes, 22 transfer RNA (tRNA) genes and one control region (D-loop), except for ND6 gene and 8 tRNA genes [tRNA-Gln, Ala, Asn, Cys, Tyr, Ser (UCN), Glu and Pro], all other genes were encoded on the H-strand. Adjacent genes overlap by a total of 23 bp in 7 different locations from 1 to 7 bp, and have spacers of a total of 70 bp in 13 different locations from 1 to 33 bp. Except the gene COX1 used GTG as the initiation codon, all PCGs of the mitochondrial genome contain the strand start codon ATG. Seven genes end with the complete stop codon TAA or TA, while the COX2, ND2, ND3, ND4 and Cytb genes terminate with an incomplete stop codon T, which are often found within the mitochondrial genomes of teleost fishes, are completed via posttranscriptional polyadenylation (Chu et al. 2013; Zhang et al. 2014). The length of 22 tRNA genes ranged from 67 to 76 bp. All tRNA genes could be folded into a typical cloverleaf structure except for tRNA-Ser (AGY), which lost the dihydrouridine arm and formed a simple loop with 12 unpaired nucleotides. The gene arrangement and transcriptional direction were the similar to those of the typical teleosts mitogenomes (Chu et al. 2013). In the base composition of mitogenome, there was 31.80% A, 27.55% C, 15.80% G, 24.85% T, and a slight AT bias of 56.65% occurred.

The sequence alignment of mitochondrial genomes between the hybrid and its female parent revealed a total of 47 variable sites in 15 genes or regions, especially, 25 sense mutations in 6 PCGs (ND1, ND2, COX1, COX2, ND4 and ND5). These sense mutations may have some influences in the function of each control region. The phylogenetic analysis (Figure 1) showed that the studied hybrid was relatively more close to *Cyprinus carpio L. mirror*, which was its female parent. All the species from *Cyprinus* genus were gathered into the same branch (Tamura et al. 2011). This result was in agreement with the conventional taxonomic relationship of these species.

**Figure 1.**
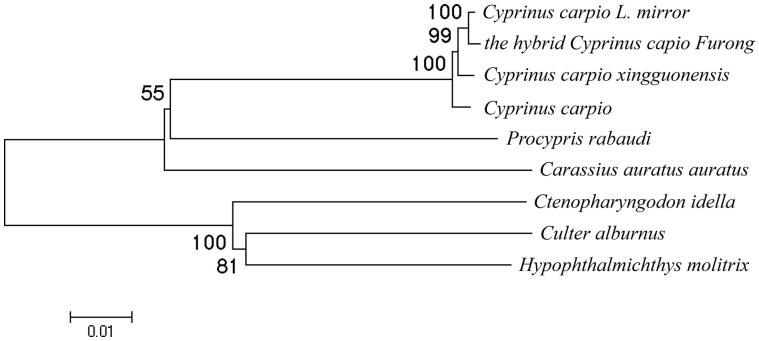
Phylogenetic relationship of the hybrid *Cyprinus capio Furong* complete mitochondrial genome DNA with other species. The phylogenetic tree was constructed by using the neighbour-joining method. The bootstrap confidence values shown at the nodes of the tree are based on 2000 bootstrap replications. Note: *Cyprinus carpio L. mirror*: KU146529; *the hybrid Cyprinus capio Furong*: KU146532; *Cyprinus carpio xingguonensis*: KU146530; *Cyprinus carpio*: KU159761; *Procypris rabaudi*: NC_011192; *Carassius auratus auratus*: KU146528; *Ctenopharyngodon idella*: NC_010288; *Culter alburnus*: NC_013616; *Hypophthalmichthys molitrix*: NC_010156.
